# Host-mimicking conditions promote *Pseudomonas aeruginosa* PA14 virulence gene expression

**DOI:** 10.3389/fmicb.2025.1557664

**Published:** 2025-04-22

**Authors:** Amber Grace, Rajnish Sahu, Donald R. Owen, Vida A. Dennis

**Affiliations:** ^1^Department of Biological Sciences, Alabama State University, Montgomery, AL, United States; ^2^Owen Biosciences Inc., Baton Rouge, LA, United States

**Keywords:** *Pseudomonas aeruginosa*, host-like media, antimicrobial susceptibility testing, virulence, RNA sequencing

## Abstract

**Background:**

*Pseudomonas aeruginosa* is a ubiquitous, opportunistic bacterium whose highly plastic genome and adaptable phenotype have yielded serious treatment challenges for immunocompromised patients. Antibiotic alternatives, such as anti-virulence therapeutics, have gained interest because they disable bacterial virulence mechanisms, thereby restoring the killing efficacy of host immunity or traditional antibiotics. Identifying successful anti-virulence therapeutics may require a paradigm shift from the decades-old antimicrobial susceptibility testing (AST) in Mueller Hinton broth to media that foster optimal virulence expression.

**Methods:**

This study evaluates the virulence gene expression and activity of *P. aeruginosa* PA14 in host-mimicking conditions, represented by Dulbecco’s Modified Eagle’s Medium (DMEM) without serum, with fetal bovine serum (FBS), or with human serum (HuS) in comparison to standard antimicrobial susceptibility testing conditions, represented by Cation-adjusted Mueller Hinton broth (CAMHB). PA14 twitching motility and pyoverdine production were evaluated under these conditions.

**Results:**

For the first time, our study reveals that culturing the highly virulent *P. aeruginosa* PA14 in host-mimicking media enhances the expression of multiple virulence therapeutic targets that are critical to host colonization and infection. RNA sequencing showed that multiple Type III Secretion (T3SS), Type I Secretion (T1SS), pyoverdine biosynthesis, uptake and efflux, and Type IV pili (T4P) initiation genes were promoted when PA14 was transitioned into host-mimicking conditions but remained unchanged when transitioned into standard AST conditions. Moreover, qPCR results disclosed that HuS and FBS delivered differential effects on the expression of membrane-associated virulence genes involved in host colonization. Our macroscopic PA14 twitching motility results aligned more closely with PA14 growth patterns than with virulence gene expression patterns. Our microtiter biofilm assay, however, revealed earlier biofilm formation in DMEM 0 than in AST conditions and both showed inhibited twitching motility in serum conditions. UV-Vis spectra showed that pyoverdine production aligned with our gene expression data, revealing higher pyoverdine production in serum conditions for planktonic PA14.

**Discussion:**

Overall, our findings support using host-mimicking conditions to improve the expression of candidate targets for anti-virulence therapeutics against *P. aeruginosa* PA14 in a planktonic state. These recommendations may be broadly applicable for antivirulence therapeutic screening against multiple bacterial species at large.

## 1 Introduction

*Pseudomonas aeruginosa* is a gram-negative, opportunistic bacillus with a highly plastic genome and phenotype filled with intrinsic resistant mechanisms (Grace et al., 2022; Diggle and Whiteley, 2020; Elfadadny et al., 2024). Over time, these characteristics, bolstered by antibiotic overuse, have increased the prevalence of multidrug-resistant, extensively drug-resistant, and pan drug-resistant *P. aeruginosa* strains on a global scale (Magiorakos et al., 2012; Kadri et al., 2018; Lister et al., 2009) Consequently, some of these strains have rendered nearly every traditional antibiotic ineffective, severely decreasing treatment options for a common nosocomial infection (Lister et al., 2009). While *P. aeruginosa* is generally harmless to healthy individuals, immuno-compromised and hospitalized patients face dire challenges if infected. Specifically, drug-resistant *P. aeruginosa* has increased mortality risks for cystic fibrosis, burn and cancer patients, and those on mechanical ventilation or with medical device implants (Liao et al., 2022; Lund-Palau et al., 2016).

Antibiotic-resistant *P. aeruginosa* indirectly or directly contributes to more than 300,000 deaths per year worldwide. This level of resistance and mortality led The World Health Organization (WHO) to designate *P. aeruginosa* as an ESKAPE (*Enterococcus faecium*, *Staphylococcus aureus*, *Klebsiella pneumoniae*, *Acinetobacter baumannii*, *Pseudomonas aeruginosa*, and *Enterobacter* species) pathogen, with a high priority need for novel therapeutics (Jesudason, 2024; Antimicrobial Resistance Collaborator, 2022). Since the WHO-issued alert, one novel therapeutic (Cefiderocol) and two β-lactam/β-lactamase inhibitor combinations (Imipenem-Cilastatin/Relebactam and Cefepime-Taniborbactam) for *P. aeruginosa* were still being evaluated in Phase III clinical trials or had received the Food and Drug Administration (FDA) approval (Reig et al., 2022). New β-lactam/β-lactamase inhibitor combinations have demonstrated improved efficacy against β-lactam resistance mechanisms possessed by *P. aeruginosa*. However, recent studies have shown that *P. aeruginosa* and other MDR pathogens have prevailed against these therapeutics in developing resistance through other mechanisms (Alonso-García et al., 2023; Sastre-Femenia et al., 2023). These therapeutic challenges bring to light the need for treatments that are less likely to perpetuate a deadly cycle of resistance.

Recently, the search for effective, long-lasting therapeutics has turned toward strategies that avoid direct killing and, instead, disable *P. aeruginosa’s* wide arsenal of virulence factors. This approach reduces selective pressures and increases susceptibility to host immunity and to traditional antibiotics (Reig et al., 2022; Liao et al., 2022). *P. aeruginosa* has numerous virulence factors, including biofilm formation, siderophores, flagella, pili, toxin secretion systems, antibiotic resistance mechanisms, etc., that could be independently inactivated or work synergistically with traditional antibiotics. Some anti-virulence therapeutics (e.g., small molecule inhibitors and antibodies) have already shown some success against *P. aeruginosa* by *in silico*, *in vitro*, and *in vivo* studies (Tacconelli et al., 2018; Kannon et al., 2022; Kang et al., 2019; Reig et al., 2022; Pendergrass and May, 2019).

Potential anti-virulence therapeutics may require new screening conditions to predict clinical appropriateness more accurately. There have recently been concerns that antimicrobial susceptibility testing (AST) methods established in the 1960’s and 1970’s to screen antibiotics may now be incompatible with newer therapeutic categories like anti-virulence drugs (Hackel et al., 2019; Ersoy et al., 2017; Belanger et al., 2022). Standard AST screening methods called for the use of Cation-adjusted Mueller-Hinton broth (CAMHB), which has, for decades, supported consistent and acceptable bacterial growth and low inhibition of therapeutic activity (Nizet, 2017). However, *P. aeruginosa* is highly adaptable to various environments and expresses different virulence and genetic factors based on the composition of its surroundings (Grace et al., 2022). The *P. aeruginosa* phenotypic profile in CAMHB is, therefore, likely to differ from the phenotypic profile in a natural infection environment.

To this point, the recently FDA-approved, siderophore-targeting Cefiderocol was found to require such an AST alteration. Traditional CAMHB makes a sufficient supply of iron available to *P. aeruginosa* and would prevent enhanced siderophore production in the bacterium. Contrarily, an actual clinical infection would promote greater siderophore production *P. aeruginosa* to thrive in an iron-limiting environment. Iron-depleted MHB also affects siderophore production– that is involved in bacterial virulence – to better mimic host infection conditions (Minandri et al., 2016). Thus, inhibiting these siderophores could effectively reduce virulence to assist in host immune system clearing or in antibiotic sensitivity restoration. An iron-depleted MHB was therefore approved to simulate a more host-mimicking environment and to allow for accurate AST of cefiderocol (Hackel et al., 2019). The above studies suggest that there could be other targetable virulence factors in *P. aeruginosa* that may not be sufficiently expressed in CAMHB.

Using the highly virulent reference strain *P. aeruginosa* PA14 as a model, the goal of our study was to determine if host-mimicking culturing conditions would promote higher expression of virulence therapeutic targets than standard AST conditions. We hypothesized that host-mimicking conditions are more appropriate for promoting the expression of virulence targets and are, therefore, more appropriate for screening anti-virulence therapeutics. We began this study by culturing PA14 in nutrient broth (NB) as a laboratory maintenance media control, CAMHB as AST media, and in Dulbecco’s Modified Eagle’s Medium (DMEM) with fetal bovine serum (FBS) as host mimicking media. For the first time, our RNA sequencing data revealed that multiple virulence factors critical for *P. aeruginosa* establishment and maintenance of infections are uniquely enhanced in media that mimic the conditions of a biological host. These same virulence factors were unchanged in CAMHB. Interestingly, the uniquely enhanced virulence systems –Type IV Pili (T4P) initiation, Type III Secretion (T3SS), Type I Secretion (T1SS) and pyoverdine—were mostly membrane-associated or were involved in extracellular secretion of virulence factors. Further analysis of select virulence genes from these categories by qPCR experiments led to our second novel finding that, when added to DMEM, fetal bovine serum (FBS) and human serum (HuS) differentially affected virulence gene expression. These results are very important in considering how serum type will affect the expression of bacterial virulence factors being evaluated for therapeutic screening. Additionally, our RNAseq studies and media growth comparisons of PA14 allowed us to confirm that these gene expression increases were independent of bacterial cell density or quorum sensing activity. This confirmation strengthens our case that virulence expression is more likely due to the environment composition.

Our study also compared T4P and pyoverdine gene expression to twitching motility and pyoverdine production, respectively. This portion of the investigation was to determine if increased virulence gene expression would result in increased virulence activity. Investigation of T4P activity through a macroscopic twitching motility assay revealed a closer association of twitching motility to PA14 growth patterns than to virulence gene expression when PA14 was in the presence of a surface. However, investigation of twitching motility with a microtiter biofilm assay revealed earlier surface attachment and biofilm formation in nutrient poor DMEM 0 than in nutrient rich CAMHB. Additionally, both investigations demonstrated reduced PA14 virulence activity in serum-containing conditions.

Contrarily, our pyoverdine production investigation closely aligned with gene expression data, as the serum containing conditions effectively produced pyoverdine in planktonic PA14. Overall, the novel data presented in this study proves that host-mimicking medium is critical for enhanced genetic expression of virulence targets in planktonic *P. aeruginosa* PA14. These results can assist in ending the therapeutic stalemate by ensuring that bacterial species can sufficiently express therapeutic target candidates in the appropriate host-mimicking conditions.

## 2 Materials and methods

### 2.1 Strain and bacterial growth conditions

*P. aeruginosa* strain PA14 was used for all experiments in this study. Apart from RNA sequencing experiments, the media used in this study included nutrient broth (NB), Cation-adjusted Mueller-Hinton broth (CAMHB), Dulbecco’s Modified Eagle’s Medium (DMEM) without serum (DMEM 0), DMEM with 10% FBS (DMEM FBS), and DMEM with 10% Human serum (HuS) (DMEM HuS). DMEM FBS and DMEM HuS were used to represent host-mimicking conditions. For the RNA sequencing experiment, only NB, CAMHB, and DMEM 10% FBS were used. To obtain bacteria for the experiments as described below, excluding the pyoverdine production experiment, planktonic *P. aeruginosa* PA14 was grown in NB at 37°C in a shaking incubator until it reached the log phase and then diluted to 2 × 10^5^ CFU/mL in one of the media or broths mentioned above and grown overnight at 37°C without shaking. For growth curves, samples were grown in triplicates in 96-well plates for 24 h, and optical density readings were taken every 2 h at 600 nm. When measuring pyoverdine production, overnight cultures were grown at 37°C with shaking.

### 2.2 *P. aeruginosa* PA14 RNA sequencing and analysis

Ribonucleic acid was extracted from PA14 grown in NB, CAMHB or DMEM FBS using the RNeasy Mini Kit (QIAGEN) and quantified using a NanoDrop One spectrophotometer (ThermoFisher). RNA sequencing was performed at the Genomics Core at the University of Alabama in Birmingham (UAB). The DNA was prepared with the NEB Next Ultra II DNA prep kit according to the manufacturer’s protocol. The RNA was prepared using the NEB NextUltra II Directional RNA kit, also in accordance with the manufacturer’s protocol. The samples were sequenced on the NovaSeq6000 using standard techniques. For analysis, raw sequence reads were first trimmed to remove primer adapters using Trim Galore (version 0.6.10; parameters used: –trim-n –trim1 –nextseq 20). The trimmed sequences were then aligned to Ensembl’s *Pseudomonas aeruginosa* (UCBPP-PA14) reference genome using STAR (version 2.7.10a; parameters used: –outReadsUnmapped Fastx –outSAMtype BAM SortedByCoordinate –outSAMattributes All –outFilterIntronMotifs RemoveNoncanonicalUnannotated) (Dobin et al., 2013). Following alignment, transcript abundances were calculated using HTSeq-count (version 2.0.2; parameters used: -m union -r pos -t exon -i gene_id -a 10 -s no -f bam) (Anders et al., 2015) Normalization and differential expression were then applied to the count files using DESeq2 (Love et al., 2014) following their default parameters in their vignette.

### 2.3 Effects of broth or media on *P. aeruginosa* PA14 virulence genes

qPCR was used to evaluate the expression of selected virulence genes after culture of PA14 in various media. RNA was extracted and the RNA purity and concentrations were determined with a NanoDrop One spectrophotometer. RNA samples (2 ng) were used to amplify selected virulence genes (T4P initiation genes: *PilY1*, *PilW*, *PilX*, *FimU*, Pyoverdine receptor and efflux genes: *FpvA*, *OpmQ*, T3SS genes: *PopD*, *PopB*, *PcrV*, Heme acquisition (T1SS) genes: *HasAp*, *HasD*, *HasE*, *HasF*, and *HasR*). The Real Time PCR amplification was carried out with the Analytik Jena Real Time PCR system and gene expression changes relative to 16S rRNA (housekeeping gene) were determined with the equation: 2^–ΔΔ*CT*^. Fold changes of mRNA gene expression were calculated against the mRNA gene expression of samples cultured in NB. Each RT-PCR experiment was performed in triplicate and repeated in three separate experiments. The results are shown as the mean + standard deviation (SD). Significance was analyzed using two-way ANOVA followed by Tukey’s multiple comparisons test. Significance between groups was considered at *p = 0.05, **p = 0.01, ***p = 0.001, ****p = 0.0001.

### 2.4 Effects of broth or media on the twitching motility of *P. aeruginosa* PA14

To determine the effects of laboratory broths or host mimicking media on twitching motility, the standard 1% LB-Lennox agar (LBA) twitching agar was infused with the broth or media types described above. Traditional 1% LBA was also used as a control (Turnbull and Whitchurch, 2014). PA14—grown to log phase in NB –was diluted to 2 × 10^5^ CFU/mL in either Lysogeny broth (LB) (used as an additional control for the 1% LB-Lennox agar) or various media as stated above. All cultures were grown overnight and 1 × 10^6^ PA14 in each media condition was added to wells within the corresponding agar type (i.e., 1 × 10^6^ PA14 in DMEM FBS culture was added to the DMEM FBS – 1% LBA agar) in petri dishes and incubated in a humidified chamber at 37°C. After 24 hours of incubation, the agar was carefully removed and the petri dishes were stained with 0.1% crystal violet to make the twitching zones apparent and washed with deionized water to remove excess crystal violet. The stained twitching zones were imaged with an Odyssey reader and the twitching zones were measured using ImageJ software. Two to four replicates of each condition were used from three separate experiments. The averages were calculated as the mean ± SD. Significance was analyzed using one-way ANOVA followed by Tukey’s multiple comparisons test.

### 2.5 Effects of broth or media on biofilm formation of *P. aeruginosa* PA14

A crystal violet biofilm assay was used to assess differences in surface attachment, time of attachment and biofilm formation of PA14 cultured in media or broth as described above. Static cultures were regrown overnight at 37°C and diluted to a 0.05 OD followed by a 1:100 dilution in each media (Coffey and Anderson, 2014). A total of five replicates (100 μL each) were dispensed into untreated 96 well-plates and incubated for 1, 2, 4, and 8 hours at 37°C. After incubation, the media were removed by gentle shaking and washed with tap water. Biofilms were stained with 0.1% crystal violet. Excess stain was removed by washing, biofilms were dried overnight and then dissolved in a solution of 70% absolute ethanol and 30% glacial acetic acid. The optical density of the dissolved biofilms was measured at 550 nm.

### 2.6 Effects of broth or media on planktonic *P. aeruginosa* PA14 pyoverdine production

Ultraviolet-visible spectroscopy (UV-Vis) was used to measure pyoverdine produced by PA14 in various media or broth (Hoegy et al., 2014). PA14 was grown for 8 hours in NB at 37°C and 200 rpm and diluted to 0.1 OD. PA14 (1 × 10^8^) was added to the culture media and grown overnight to stationary phase for 15 hours at 37°C and 200 rpm. Each culture was diluted to 0.2 OD, and 100 μL was completed to 1 mL with 50 mM pyridine–acetic acid buffer (pH 5.0). The resulting spectra were read on a NanoDrop One spectrophotometer (Thermo Scientific).

### 2.7 Iron content determination of various media and broth

We performed an iron assay (BioAssay Systems QuantiChrom Iron Assay Kit DIFE-250) to quantify the amount of iron (Fe^2+^) in NB, CAMHB, DMEM 0, DMEM FBS, and DMEM HuS. The assay was conducted as described in the manufacturer’s protocol. A blank was run for all samples and the optical density was read at 590 nm.

## 3 Results

### 3.1 Host mimicking and AST conditions differentially affect the function of *P. aeruginosa* PA14 at the transcriptional level

A comprehensive assessment of *P. aeruginosa* transcriptional behavior under host mimicking and AST conditions is essential to determining which media best supports adequate expression of candidate therapeutic targets. Such studies may also provide a thorough analysis of the *in vitro* behavior of *P. aeruginosa* to compare to known clinical infection data (Cornforth et al., 2018). Thus, we sought to investigate the transcriptomic profiles of PA14 after it transitions from maintenance media into either AST or host-mimicking media. Reportedly, many virulence factors are induced when *P. aeruginosa* transitions from the environment to the host (Qin et al., 2022).

To compare *P. aeruginosa* PA14 transcriptional profiles under different culture conditions, we performed RNA sequencing after PA14 was cultured in NB, a common maintenance media and then transitioned into NB, as a control; CAMHB, a standard AST media, and DMEM FBS, host mimicking media. Comparative analyses of the culturing conditions were made—DMEM FBS vs. NB, CAMHB vs. NB, and DMEM FBS vs. CAMHB. Within these groups, we looked for uniquely expressed PA14 genes—genes with a ≥ 2-fold change and a *p*-value of ≥ 0.05 that are promoted or repressed in one culturing condition and not in the other two.

Through the analysis of 5,952 genes, we found 166 genes uniquely expressed when PA14 was transitioned from NB to DMEM FBS but not when PA14 was transitioned from NB to CAMHB. Functionally, genes categorized as small molecules were most affected under host-mimicking conditions. Of the well-characterized genes, *fpvA*, *HasR* and *HasAp* had the highest fold increases— 4.95, 5.45, and 15.05, respectively (Supplementary Table 1). The significant increase in the expression of these genes points to increased iron siderophore (*fpvA*) and hemophore (*HasR* and *HasAP*) activity. Notably, these factors have been directly or indirectly assessed as therapeutic targets (Centola et al., 2020; Hamad et al., 2022; Sen-Kilic et al., 2019; Imperi et al., 2013; Kirienko et al., 2016).

**FIGURE 1:**
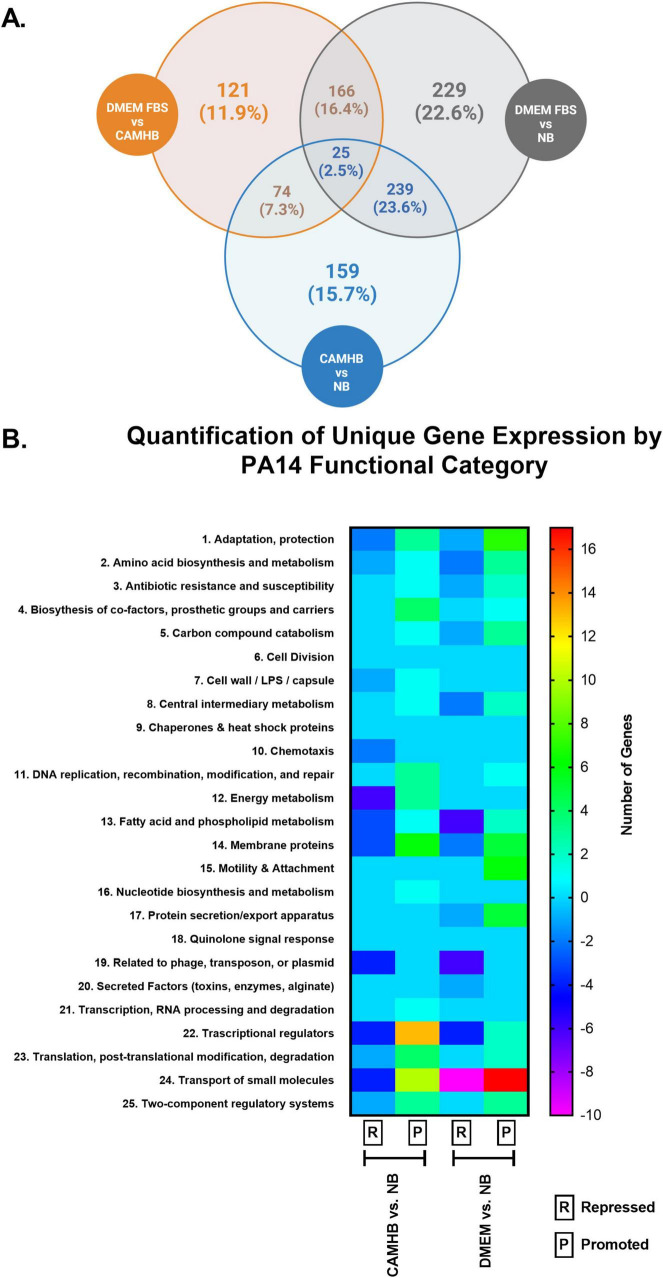
Comparison of unique gene expression of PA14 in laboratory maintenance, antimicrobial susceptibility testing (AST) and host-mimicking culture conditions. *P. aeruginosa* PA14 was grown to log phase in nutrient broth (NB), and then 2 × 105/mL PA14 was transitioned to NB, CAMHB, and DMEM FBS for overnight incubation. RNA was extracted and used for RNA sequencing. **(A)** Venn diagram shows the number and percentages of PA14 genes uniquely expressed when PA14 was cultured in DMEM FBS, CAMHB, and NB. Unique expression is defined as a = 2-fold change and a *p*-value = 0.05. This figure was created with Biorender. **(B)** The heat map shows the number of repressed and promoted genes in each functional category when CAMHB was compared to NB and when DMEM FBS was compared to NB. The RNA-sequencing experiment was repeated three times.

A separate 159 genes were uniquely expressed when PA14 was transitioned from NB to CAMHB (Figure 1A and Supplementary Table 1). Under these AST conditions, PA14 transcriptional regulators were most affected. Unlike small molecule gene expressions under host-mimicking conditions, the fold changes were comparatively low and ranged from -2.09 (PA14_37660: LysR-type transcriptional regulator) to 3.63 (PA14_55550: probable sigma-70 factor, ECF subfamily). AST conditions also repressed expression of *nosR* (-2.65), which is involved in denitrification (Arai et al., 2003), and *glmR* (-2.54). *GlmR* is thought to be involved in metabolizing amino sugars (Ramos-Aires et al., 2004). Interestingly, when *glmR* is inactive, *P. aeruginosa*’s susceptibility to antibiotics, like aminoglycosides and vancomycin, increases (Ramos-Aires et al., 2004). These results confirm that the transition of *P. aeruginosa* PA14 into AST conditions does not promote expression of the host infection and colonization factors seen when PA14 is transitioned into host-mimicking media.

When we compared the effects of host-mimicking and AST conditions on the 28 functional categories for PA14, we observed that some functional categories were exclusively affected under each condition (Figure 1B) (PA14 Gene Annotations, 2005). Genes within the cell wall/LPS/capsule, chemotaxis, energy metabolism, nucleotide biosynthesis and metabolism, transcription and RNA processing, and degradation functional categories were exclusively promoted or repressed under standard AST conditions when compared to the NB control. In contrast, motility and attachment, protein secretion/export apparatus, and secreted factors (toxins, enzymes, alginate) functional categories were exclusively affected under host-mimicking conditions when compared to the NB control. The functional categories exclusively affected under host-mimicking conditions are notable because they are involved in host colonization and infection. These findings, again, support host-mimicking media as choice conditions for therapeutic screening against host colonization and infection factors.

**FIGURE 2:**
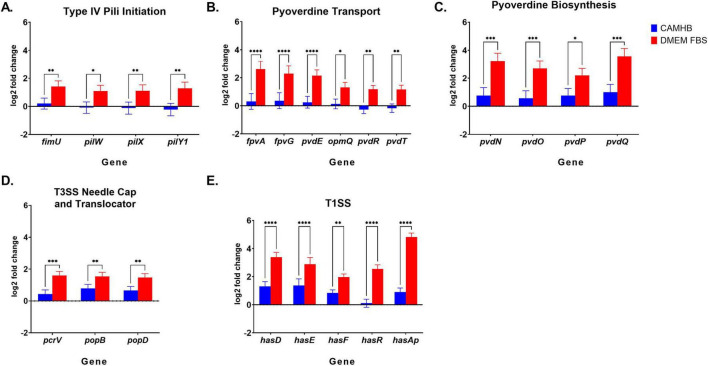
Changes in PA14 virulence RNA expression levels between CAMHB and DMEM FBS. RNA-sequencing is the same as described above in Figure 1 legend. **(A)** Type IV pili initiation, **(B)** Pyoverdine transport, **(C)** Pyoverdine biosynthesis, **(D)** Type III Secretion System (T3SS) needle cap and translocator and **(E)** Type I (T1SS) gene expression of PA14 in CAMHB and DMEM FBS. Both CAMHB and DMEM FBS are normalized against PA14 gene expression in NB. All genes shown, except *PopD* and *PopB*, were selected from the 166 genes with unique fold increases (≥2, *p*-value of = 0.05) for PA14 expression in DMEM FBS compared to PA14 expression in CAMHB. *PopD* and *PopB* showed unique fold changes (≥2, *p*-value of = 0.05) when PA14 expression in DMEM FBS was compared to that in NB. The error shown is the lfcSE, or standard error of the log2 Fold Change estimate. The RNA-sequencing experiment was repeated three times. Significance between PA14 gene expression levels in CAMHB and DMEM FBS was considered at *p≤0.05, **p≤0.01, ***p≤0.001, ****p≤0.0001.

For anti-virulence therapeutics to be effective, the virulence target must be sufficiently expressed by the bacterium. For the first time, our RNA transcriptome data revealed that the expression of multiple PA14 virulence categories was uniquely promoted in DMEM FBS. The first group of virulence genes with unique fold change increases in DMEM FBS includes Type IV pili (T4P) initiation genes *FimU, PilW, PilX*, and *PilY1*. *FimU, PilW*, and *PilX* form part of the operon that encodes minor pilins (FimU-PilVWXE) and is involved in pilus initiation (Leighton et al., 2015). PilY1 forms part of the pilin subunit with FimU-PilVWXE and is essential for pilus formation (Leighton et al., 2015). Log2fold changes were significantly higher (*FimU, PilX, PilY1*, *p* = 0.01; *PilW*, *p* = 0.05) for PA14 when DMEM FBS was compared to NB as opposed to expression of the same genes when CAMHB was compared to NB (Figure 2). *PilV* and *PilE* were not significantly promoted in DMEM FBS compared to CAMHB orNB (Supplementary Tables 2, 3). Notably, the expression of the major elongation pilin subunit *PilA* did not significantly change between tested conditions in this experiment (Supplementary Table 2). Increased expression of *FimU, PilW, PilX*, and *PilY1* suggests that the transition from the NB laboratory maintenance media to host-mimicking conditions DMEM FBS causes PA14 to recognize the latter as an environment in which surface attachment and motility capabilities are more necessary or advantageous. Standard AST conditions do not produce a similar effect in *P. aeruginosa* PA14.

Culturing *P. aeruginosa* PA14 in DMEM FBS also led to the promotion of pyoverdine synthesis gene expression that involved precursor genes *PvdN*, *PvdO*, *PvdP* and *PvdQ* for PVDI (Figure 2C). PVDI is a key siderophore and virulence factor used to acquire iron in the iron-scarce host environment (Ghssein and Ezzeddine, 2022). The log2fold changes for genes coding for the membrane components responsible for iron uptake (*FpvA, p* = 0.0001), inner membrane iron release (*FpvG, p* = 0.0001), iron cytoplasm transport (*FpvE*, *p* = 0.0001) and efflux of PVD1 (*PvdT, p* = 0.01, *PvdR, p* = 0.01, and *OpmQ, p* = 0.05) increased significantly when DMEM FBS was compared to NB as opposed to expression of the same genes when CAMHB was compared to NB (Ghssein and Ezzeddine, 2022; Figure 2B). Interestingly, the fold changes in expression of *FpvA* for PVD-Fe^3+^ complexes was approximately twice that of PVDI genes involved in efflux (PvdRT-OpmQ) (Supplementary Table 1). Also of interest, *P. aeruginosa’s* secondary iron acquisition siderophore pyochelin was not uniquely promoted in host-mimicking conditions. Pyochelin operons *pchABCD* and *pchREFG* were promoted approximately 2 to 5-fold in both DMEM FBS and CAMHB when compared to NB (Supplementary Tables 3, 4). The increase in pyoverdine expression seen with PA14 in DMEM FBS when compared to CAMHB may indicate that the host-mimicking conditions used in this study are more appropriate for evaluating siderophore-targeting therapeutics.

Other uniquely promoted PA14 genes observed in DMEM FBS host-mimicking as opposed to standard AST conditions were those involved in iron acquisition such as the TISS genes (include the heme acquisition (Has) system genes *HasD*, *HasE*, *HasF*, *HasR*, and *HasAp*). Notably, the gene coding for hemophore *HasAp* was the most up-regulated virulence gene uniquely promoted in DMEM FBS (Figure 2E).

PA14 in DMEM FBS also led to the unique promotion of T3SS translocator gene expression. Expression of *PcrV*, which forms the base of the translocator apparatus that injects exoenzymes into host cells, increased significantly (*p* = 0.001) (Figure 2D) (Horna and Ruiz, 2021). Assembled atop PcrV are PopD and PopB, which were not markedly enhanced in DMEM FBS vs. CAMHB (Supplementary Table 1), but expression increase significantly *p* = 0.01, respectively, in DMEM FBS vs. NB (Figure 2D). The fold change for *PcrV* expression when DMEM FBS was compared to NB was 3.4 (Supplementary Table 1). Like other virulence genes discussed in this section, the increased expression in DMEM FBS may be a more suitable therapeutic testing environment when targeting PcrV.

### 3.2 FBS and HuS differentially affect the expression of T3SS, iron acquisition and pili initiation genes

Since *P. aeruginosa* virulence targets are usually assessed for human therapeutic design, it is critical to know whether FBS would affect virulence expression similarly to HuS. This comparison is necessary, as FBS is the most used animal serum for *in vitro* cell culture experiments. Additionally, FBS supports the satisfactory growth of animal and human cells and is less expensive than HuS (Fang et al., 2017). It is also important to identify whether the serum in DMEM is responsible for the increases in PA14 virulence gene expression. To determine the effect of serum on PA14 membrane-associated virulence gene expression, we cultured PA14 as done for RNA sequencing studies, but with the following additional conditions: DMEM with no serum (DMEM 0) and DMEM HuS. Our experiment is the first to compare FBS and HuS on virulence gene expression in PA14.

**FIGURE 3:**
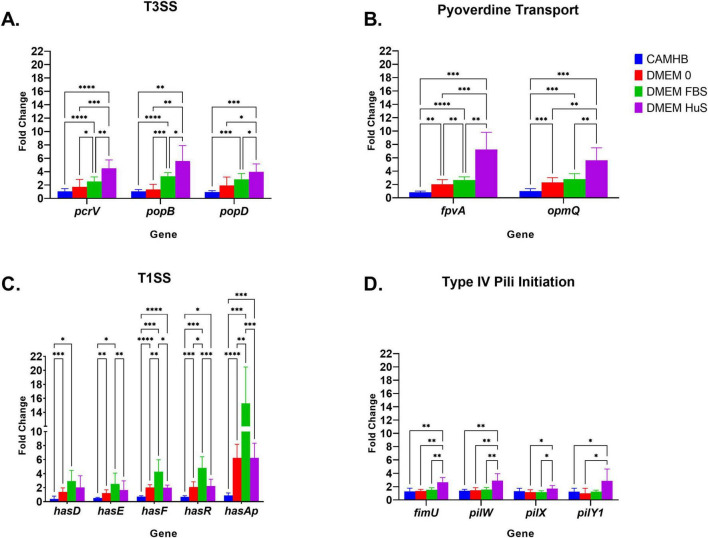
Comparison of RNA expression for select virulence genes. RNA sequencing is the same as described above in Figure 1 legend. **(A)** T3SS, **(B)** Pyoverdine Siderophore, **(C)** T1SS and **(D)** Type IV Pili initiation PA14 gene expression in CAMHB, DMEM 0, DMEM FBS and DMEM HuS. The fold change is against virulence gene expression in nutrient broth (NB). Each bar represents the mean ± SD of triplicate samples from three separate experiments. Significance was analyzed using two-way ANOVA followed by Tukey’s multiple comparisons test. Significance between groups was considered at *p = 0.05, **p = 0.01, ***p = 0.001, ****p = 0.0001. .

In this experiment, we extracted RNA and evaluated the effects of the various culture conditions on T3SS genes (*PcrV, PopD* and *PopB*); Pyoverdine uptake and efflux genes (*FpvA* and *OpmQ*); T1SS genes (*HasD, HasE, HasF, HasR*, and *HasAp*), and T4P minor pili genes (*FimU, PilW, PilX*, and *PilY1*). We chose to focus on these genes because the RNA sequencing analysis revealed that multiple membrane-associated virulence genes were uniquely affected. *HasAp* was the only extracellular gene evaluated here. The fold changes shown in Figure 3 are against PA14 expression levels in NB.

**FIGURE 4:**
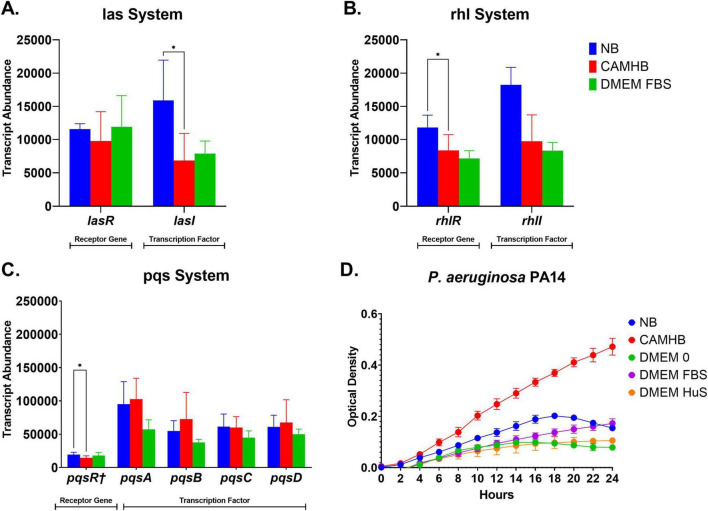
*P aeruginosa* PA14 expression of quorum sensing genes and growth curves in maintenance, antimicrobial susceptibility testing (AST) and host mimicking conditions. **(A–C)** Bar graphs show comparative expressions of *las*, *rhl*, and *pqs* receptor and transcription factor genes for PA14 in nutrient broth (NB), CAMHB, and DMEM FBS. Each bar represents the mean ± SD of samples from three separate RNA seq experiments. Significance was analyzed using one-way ANOVA followed by Tukey’s multiple comparisons test. Significance between groups was considered at *p = 0.05. **(D)** Growth curves show the growth pattern of PA14 in maintenance, AST and host-mimicking conditions. PA14 was grown overnight in NB and then transitioned into NB, CAMHB, DMEM 0, DMEM FBS, or DMEM HuS. All samples were then grown in triplicates in 96 well plates for 24 h and optical density readings at 600 nm were taken every 2 h. Data ± SD shows results from three experiments.

The expression pattern for the T3SS genes *PcrV*, *PopD* and *PopB* was like that derived from the RNA sequencing data. CAMHB yielded no significant change in expression of these genes from NB conditions, while DMEM FBS increased expression between 2- and 4-fold (*PcrV, PopB*: *p* = 0.0001, *PopD*: *p* = 0.001). Interestingly, this expression was more pronounced when PA14 was exposed to DMEM HuS. The expressions of PA14 *PcrV, PopB, and PopD* increased approximately 4 to 8-fold and were significantly higher in DMEM HuS over that in CAMHB (*PcrV*: *p* = 0.0001*, PopB*: *p* = 0.01 and *PopD*: *p* = 0.001) (Figure 4A). These results show that serum types differentially affect the expression of virulent genes. Additionally, PA14 expression in DMEM 0 was not significantly higher than in CAMHB. This suggests that the presence of serum does indeed play a role in promoting virulence gene expression.

The expression levels for PA14 pyoverdine receptor gene *FpvA* and efflux pump gene *OpmQ* were significantly higher in DMEM HuS than in CAMHB (FpvA, *p* = 0.0001, OpmQ, *p* = 0.01), and increased more than 5-fold. The expression of *FpvA* and *OpmQ* increased at least 2-fold when PA14 was cultured in DMEM 0 and DMEM FBS. However, the transition of PA14 from NB to CAMHB yielded no fold increase in expression. These results prove that the host-mimicking conditions are effective in promoting pyoverdine receptor and efflux expression (Figure 3B).

The qPCR results for the Has (T1SS) genes aligned with our RNA sequencing data. However, a very important finding was that Has (T1SS) genes were the only gene category evaluated in which DMEM FBS yielded the highest fold changes in PA14. Also of note, no significant differences were seen in Has gene expression when PA14 was grown in DMEM HuS and DMEM 0. Most evident was the significant fold increase of the *HasAp* gene in DMEM 0 (*p* = 0.0001), DMEM FBS (*p* = 0.001), and DMEM HuS (*p* = 0.001) compared to that in CAMHB. Fold change of PA14 membrane associated genes *HasD*, *HasF*, *HasR*, and *HasE* increased at least 2-fold (*HasD*, *p* = 0.05; *HasE*, *p* = 0.05; *HasF* and *HasR*, *p* = 0.001) in the DMEM FBS condition (Figure 3C). Although the reason is currently unclear, these novel findings confirm that serum types do differentially affect the expression of membrane-associated PA14 virulence genes.

When compared to expression in CAMHB, we also observed that DMEM HuS was the only condition that yielded a significant fold increase in PA14 T4P minor gene expression for *FimU* (*p* = 0.01), *PilW* (*p* = 0.01), and *PilY1* (*p* = 0.05), which contrasts the other virulence gene categories discussed above (Figure 4D).

### 3.3 Quorum sensing or increased density do not drive the increased *P. aeruginosa* PA14 expression of select virulence genes

A significant portion of *P. aeruginosa* virulence is controlled by quorum sensing (QS) mechanisms, which are activated by increasing cell densities and progression through the bacterial growth phases (Liao et al., 2022; Arevalo-Ferro et al., 2003). Pyoverdine biosynthesis, pili activity (specifically that of *PilY1*) and *HasAp* are positively associated with quorum sensing, although they are not directly controlled by it (Arevalo-Ferro et al., 2003; Siryaporn et al., 2014; Stintzi et al., 1998). T3SS is the only system evaluated in the study that is negatively impacted by quorum sensing (Pena et al., 2019). The promotion of PA14 genes involved in iron acquisition, pili initiation, and T3SS under host-mimicking conditions led us to investigate whether specific PA14 cell densities and quorum sensing expression played a role in gene promotion. We hypothesized that higher PA14 cell densities were not responsible for the increase in virulence gene expression observed under host-mimicking conditions. To test our hypothesis, we cultured PA14 in NB, CAMHB, DMEM 0, DMEM FBS, and DMEM HuS using the same parameters from our qPCR experiment. Optical densities were recorded at 600 nm every two hours over a 24 hours period. Interestingly, we found that PA14 in all host-mimicking conditions—DMEM 0, DMEM FBS, and DMEM HuS—had lower optical densities than in the AST (Figure 4D). This is important because the lower optical density of PA14 in DMEM FBS and DMEM HuS yielded higher virulence gene expressions than PA14 in NB and in CAMHB. Of significance, PA14 in CAMHB had the highest optical density over a 24 hours period but did not promote increased expression of the tested virulence genes.

Since PA14 expressed higher virulence genes in conditions with lower bacterial growth, we assessed whether significant differences in quorum sensing gene expression were perhaps responsible. We compared the expression of the transcription and receptor genes for the *las*, *rhl*, and *pqs* quorum sensing systems for PA14 (Figures 4A–C). In contrast to the expression of all our focus genes, we found that, among the NB, CAMHB, and DMEM FBS conditions, PA14 yielded significantly higher (*p* = 0.05) expression of *lasI*, *rhlR*, and *pqsR*† in NB than in CAMHB. This finding is important, considering that the previously evaluated virulence genes in both culturing conditions are statistically the same in terms of significance (Figure 3). More importantly, the expression of the same quorum sensing genes between CAMHB and DMEM FBS were statistically similar (Figures 4A–C). This contrasts with the significantly higher virulence gene expression for PA14 in DMEM FBS than in CAMHB (Figure 3). These results suggest, from a genetic standpoint at least, that increased expressions of the evaluated iron acquisition, pili initiation, and T3SS genes are not initiated by higher cell density nor by quorum sensing activity.

### 3.4 *P. aeruginosa* PA14 twitching motility patterns in different culture conditions differ significantly from virulence expression

Our RNAseq studies revealed a novel finding that host-mimicking conditions only partially promote the expression of select membrane-associated virulence systems (Figure 3 and Supplementary Table 2). This raises the question of whether the partially promoted virulence systems would produce a significant change in the actual virulence activity of PA14. To address this inquiry, we continued studies with T4P by evaluating the twitching motility of PA14 in maintenance, AST, and host-mimicking conditions. We cultured PA14 in LB (as an additional control), NB, CAMHB, DMEM 0, DMEM FBS, and DMEM HuS as performed in our qPCR studies. We then used a macroscopic twitching motility assay to evaluate the effects of laboratory maintenance, AST, and host-mimicking conditions on PA14 twitching motility. In addition to the standard 1% twitching agar (LBA) control, we created modified twitching agar conditions by infusing concentrations of NB, CAMHB, DMEM 0, DMEM FBS, and DMEM HuS like those used in previous experiments into 1% twitching agar. These modifications would allow PA14 to remain in the maintenance, AST, and host-mimicking conditions for the 24 hours incubation period.

**FIGURE 5:**
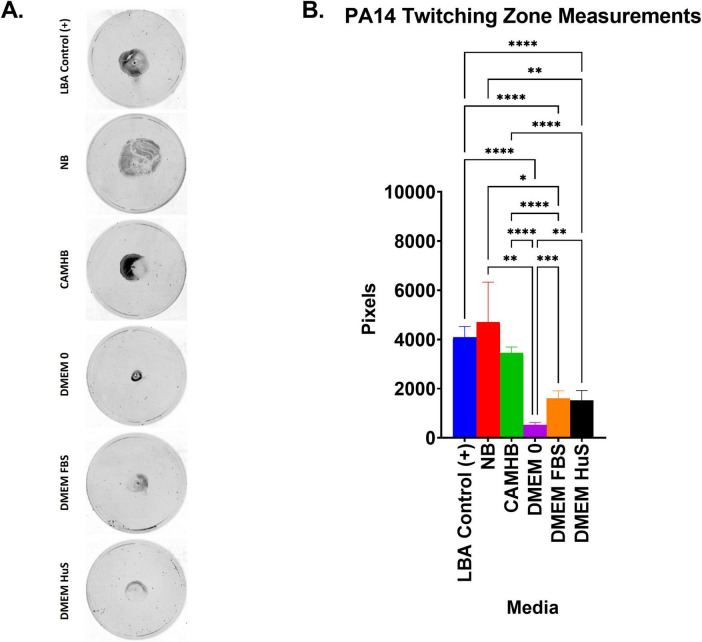
Twitching motility zone sizes compared to PA14 growth in different media. **(A)** Graph showing the area measured in pixels for PA14 in 1% LBA agar containing no additional media. Each bar represents the mean ± SD of 2–4 replicates from three experiments. Significance was analyzed using one-way ANOVA followed by Tukey’s multiple comparisons test. Significance between groups was considered at *p ≤ 0.05, **p ≤ 0.01, ***p ≤ 0.001, ****p ≤ 0.0001. Twitching zone areas were determined with ImageJ. **(B)** Images show the twitching zone size and morphology after staining with 0.1% crystal violet. **(B)** Is a representative image from three experiments.

**FIGURE 6:**
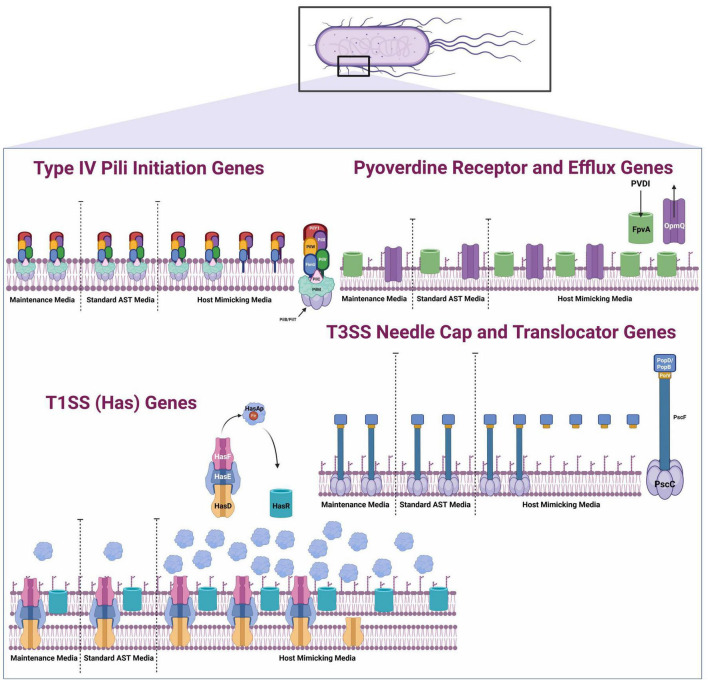
Schematic of select membrane-associated and extracellular *P. aeruginosa* PA14 virulence expression in different culturing conditions. RNAseq summary schematic illustrates the differential PA14 virulence gene expression when PA14 is transitioned from maintenance media (NB) to Standard AST media (CAMHB) and to host mimicking media (DMEM FBS) (Nguyen et al., 2015; Marko et al., 2018; Treuner-Lange et al., 2020; Bonneau et al., 2020; Cornelis, 2010; de Sousa et al., 2021; Cornelis and Dingemans, 2013; Reinhart and Oglesby-Sherrouse, 2016; Qin et al., 2022; Matteï et al., 2011; Jacobsen et al., 2020). This figure was created with Biorender.

The most significant result from our twitching motility study was that PA14 in 1% LBA, NB-1% LBA, and CAMHB-1% LBA produced larger twitching zones when compared to PA14 twitching zones in DMEM 0-1% LBA, DMEM FBS-1% LBA, and DMEM HuS-1% LBA (Figures 5A, B). This is of interest because PA14 in NB did not produce the highest growth, nor did it produce the highest virulence in any select category (Figures 3, 4D). Aside from the twitching zone produced by PA14 in NB-1%LBA, the twitching zone sizes for PA14 in CAMHB-1% LBA, DMEM 0-1% LBA, DMEM FBS-1% LBA and DMEM HuS-1% LBA aligned closely with the growth pattern seen for PA14 in these media. The differences in twitching motility compared to those seen with T4P initiation gene expression studies suggest that promotion of some T4P virulence genes when PA14 is in a planktonic state may not directly predict increased twitching motility. How these differences in gene expression (summarized in the Figure 6 schematic) and phenotypic expression would impact the success of host-mimicking media for anti-virulence therapeutic screening are currently unknown.

### 3.5 DMEM 0 promotes earlier attachment than CAMHB during PA14 biofilm formation

**FIGURE 7:**
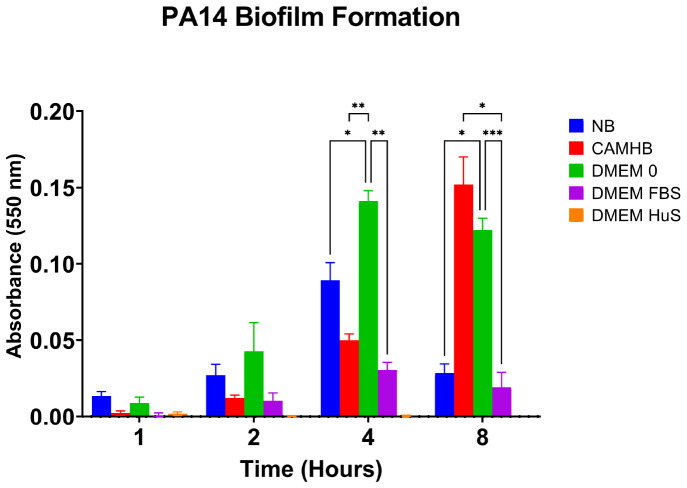
Antimicrobial susceptibility testing (AST) and host mimicking conditions affect PA14 biofilm formation. The bar graph shows the absorbance values indicating biofilm formation of PA14 in various culture conditions. Each bar represents the mean ± SEM of five replicates from three separate experiments. Absorbance was measured at 550 nm. Significance was analyzed using two-way ANOVA followed by Tukey’s multiple comparisons test. Significance within the time points were considered at *p = 0.05, **p = 0.01, ***p = 0.001.

Since twitching motility is required for biofilm formation, we also assessed T4P activity with a crystal violet microtiter biofilm assay (O’Toole and Kolter, 1998). We aimed to determine whether AST or host-mimicking conditions would promote earlier biofilm formation and surface attachment at different time points. We observed that biofilm formation was not significantly different between culture conditions until 4 hours (Figure 7) where PA14 in DMEM 0 produced significantly more biofilm than PA14 in NB (*p* = 0.05), CAMHB (*p* = 0.01), and DMEM FBS (*p* = 0.01). Interestingly, PA14 in DMEM HuS did not form biofilms at any time point. However, by 8 hours, biofilm formation increased in the CAMHB to a similar level as PA14 in DMEM 0. Biofilm formation in NB decreased from 4 to 8 hours, and DMEM FBS maintained similar levels of biofilm formation between 4 and 8 hours. When comparing host mimicking and AST conditions, these results suggest earlier surface attachment in DMEM 0 than in CAMHB. Of interest, other studies have used serum-free media to mimic biofilm-promoting *in vivo* conditions (Singh et al., 2021). Reduced biofilm formation in DMEM FBS and absent of biofilm formation in DMEM HuS are expected, as serum has been shown to reduce biofilm formation on plastic surfaces (Hammond et al., 2010).

### 3.6 Host-mimicking conditions promote pyoverdine production in planktonic PA14

**FIGURE 8:**
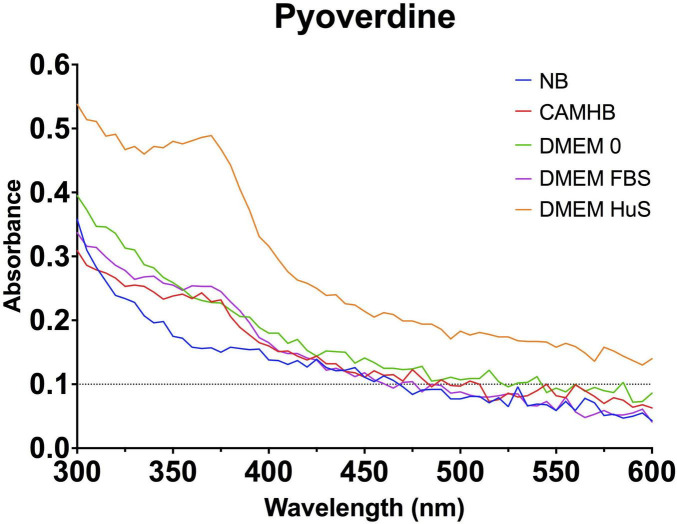
Host-mimicking conditions produce the highest amount of pyoverdine under planktonic conditions. Line graph shows the absorbance of PA14 in various culture conditions between 300 and 600 nm. Pyoverdine has a maximum absorption at approximately 380 nm (Hoegy et al., 2014). The line graph is representative of three separate experiments.

The differences observed between PA14 T4P expression and twitching motility prompted virulence investigation into PA14 pyoverdine production. Here, we determined if pyoverdine production patterns of PA14 in NB, AST, and host-mimicking media mirrored gene expression patterns when grown under planktonic conditions. Studies show that pyoverdine production increases during biofilm formation. However, disturbances to biofilms can reduce pyoverdine production (Quinn et al., 2021). In this study, we subjected all cultures to consistent shaking incubation, promoting the planktonic condition used in antimicrobial screening. We found that PA14 in host mimicking conditions (DMEM FBS and DMEM HuS), produced the highest concentration of pyoverdine, indicated by the absorbance peak near 375 nm. While we did not directly measure gene expression of PVDI precursors of PA14 in DMEM HuS, *fpvA* and *OpmQ* receptor expression levels were highest for PA14 in DMEM HuS. Our qPCR data showed f*pvA* expression for PA14 in DMEM FBS was statistically higher than that in DMEM 0 (*p* = 0.01) and CAMHB (*p* = 0.0001), data (Figure 8). Additionally, the pyoverdine spectra for PA14 in DMEM FBS and CAMHB directly align with the RNAseq results. Interestingly, little or no pyoverdine was detected in DMEM 0, CAMHB, or NB culture conditions. Overall, gene expressions for pyoverdine precursors and receptors appear to be parallel and predictive of actual virulence expression. Importantly, PA14 in host-mimicking DMEM HuS proved to be the strongest producer of pyoverdine among tested culture conditions.

We also performed an iron assay to quantify iron in all 5 media conditions as a correlation for pyoverdine production. As shown in Table 1, DMEM FBS contained the highest iron content (17.1481 ± 1.4706 μg/dL) and the second highest amount of pyoverdine according to spectral data. DMEM HuS contained the second lowest amount of iron (6.0185 ± 0.9876 μg/dL) but produced the highest amount of pyoverdine. DMEM 0 contained negligible iron (0.8889 ± 1.4790 μg/dL) with an undetectable amount of pyoverdine. NB and CAMHB contained 14.7037 ± 1.5785 μg/dL and 14.0185 ± 1.3830 μg/dL of iron, respectively, but also had undetectable pyoverdine. While iron starvation is the most common stimulator of pyoverdine production, these results do align with our RNAseq and qPCR data, which show increased pyoverdine-associated gene expression in serum-containing conditions (Butaitë et al., 2018). As such, DMEM HuS appears to be an effective pyoverdine producer under planktonic conditions.

**TABLE 1:** Iron concentrations in various media and broth.

DMEM 0	DMEM HuS	CAMHB	NB	DMEM FBS
0.8889 ± 1.4790 μg/dL	6.0185± 0.9876 μg/dL	14.0185± 1.3830 μg/Dl	14.7037± 1.5785 μg/dL	17.1481± 1.4706 μg/dL

## 4 Discussion

Overall, our study brings to light a critical discovery that *P. aeruginosa* PA14 has diverging transcriptome responses to AST (CAMHB) and host-mimicking (DMEM with serum) environments when transitioned from laboratory conditions (NB). As represented by the Figure 6 schematic, expression of some important PA14 host colonization and infection virulence systems does not change significantly from laboratory conditions to AST conditions. This stagnant expression suggests that the bacterium does not recognize an increased need to express host colonization virulence factors in the AST environment. However, the significant (*p* = 0.05) promotion of PA14 membrane associated virulence systems in host-mimicking media more closely aligns with the bacterium’s behavior when it transitions from the environment to a susceptible host (Damron et al., 2016; Belanger et al., 2020; Kruczek et al., 2012; Cornforth et al., 2020; Belanger et al., 2022). These observations support our hypothesis that *P. aeruginosa* expressing higher levels of key host colonization virulence systems in host-mimicking media would make the media a more appropriate choice for the screening of anti-virulence therapeutics.

Overall, our qPCR data aligned well with our RNAseq data for CAMHB and DMEM FBS. T4P was the only discordance, as qPCR expression for PA14 in DMEM FBS did not increase as it did in the RNAseq results. However, differences in analysis and low fold change could account for this discrepancy (Coenye, 2021). Our RNAseq and qPCR data confirmed that the *P. aeruginosa* PA14 virulence systems (T3SS, T1SS, Pyoverdine biosynthesis, uptake and efflux, and T4P initiation) contain genes coding for virulence factors that have already been evaluated as therapeutic targets. T3SS *PcrV*, which forms the base of the translocator apparatus for the secretion system and may assist in the assembly of *PopD* and *PopB* gene products, has been investigated *in vitro* and in clinical trials as an antibody target and vaccine candidate (Horna and Ruiz, 2021; Hauser, 2009; Wang et al., 2023; Long et al., 2024; Wan et al., 2019). The promotion of pyoverdine biosynthesis gene expression (*PvdN*, *PvdO*, *PvdP*, *PvdQ*, and *PvdE*) under host-mimicking conditions holds therapeutic significance as well. Pyoverdine inhibitors have been shown to decrease virulence and increase survival of *Caenorhabditis elegans* infected with MDR *P. aeruginosa* (Kang et al., 2019). *In vitro*, multiple pyoverdine inhibitors have been screened in media that promote pyoverdine expression. Imperi et al. (2013) screened compounds for pyoverdine inhibition in a low-iron trypticase soy broth dialysate (TSBD). Wurst et al. (2014) used SM9 media to identify *PvdQ* inhibitors (Imperi et al., 2013). While low iron-containing media effectivity promotes pyoverdine expression, they do not completely mimic the host environment, which contains factors that affect the bacteria’s phenotype (Vyas et al., 2022). This could lead to a bias in virulence expression that could skew the true effect of potential therapeutics. Therefore, inhibitors could be more accurate when *P. aeruginosa* is screened under holistic host-mimicking conditions.

Additionally, standard CAMHB was found to prevent sufficient expression of iron siderophores in gram-negative bacteria, skewing minimum inhibitory concentrations (MICs) of the siderophore cephalosporin, cefiderocol (Simner and Patel, 2020; Hackel et al., 2019). It is then possible that screening pyoverdine inhibitors under AST conditions may be less effective than if done in host-mimicking media. Adding significance to the increased expression of PA14 T1SS (Has) genes seen in our study, *HasAp* has also been evaluated as a vaccine target *in silico* and *in vivo*. Thus, conditions in which the hemophore is more highly expressed may be more appropriate for continued drug targeting (Hamad et al., 2022). The promotion of PilY1 in host mimicking conditions may also be ideal, as PilY1 has been identified as a desirable target for anti-virulence therapeutics (Siryaporn et al., 2014; Duménil, 2019; Ozcan et al., 2023). Also, loss of PilY1 prevented infection and killing in an amoeba host (Siryaporn et al., 2014). These investigations are highly relevant, as they demonstrate the need for optimal expression of the PA14 targets promoted under host mimicking conditions in this study.

Even more opportunities exist for anti-virulent therapeutics to work synergistically with traditional antibiotics to resensitize resistant bacteria. Many anti-virulence therapeutic studies use media that enhance the target of interest (Imperi et al., 2013). Combination therapy with antibiotics may still be most effective and accurate in host-mimicking media. Rezzoagli et al. (2020) used target-appropriate media to test combinations of antibiotics and anti-virulence therapeutics against *P. aeruginosa*. Additionally, studies have found that host-mimicking media is more predictive of clinical outcomes than AST conditions (Ersoy et al., 2017).

This optimal expression of previously discussed virulence targets may depend on the type of serum used to provide a host-mimicking environment for PA14 according to our qPCR experiment. For the first time, we reveal FBS and HuS differentially affect PA14 virulence gene expression. HuS was more effective in increasing T3SS translocator, pyoverdine receptor, and T4P gene expression, while FBS was more effective in promoting expression of all T1SS (Has) genes tested. Currently, the reason for the differences remains unclear. *P. aeruginosa* can infect both humans and animals, and both the FBS and HuS used in this experiment were heat inactivated. Further research could determine if differences in blood typing—which is unknown for FBS and is AB for HuS we used—could play a role. Additionally, small molecule components of FBS have been shown to impact experiments involving immune cells (Liu et al., 2023). A possibility also exists that components present in FBS and absent in HuS could stimulate a different response in *P. aeruginosa*. In the field of antimicrobials, these results will compel future investigators to consider whether serum yielding the highest virulence gene expression or the serum most like the intended host should be used in virulence targeting studies.

Our investigation also raised considerations on the impact of growth on virulence expression versus the impact of growth on actual virulence of PA14 in laboratory maintenance, AST and host-mimicking conditions. Interestingly, our RNAseq data show that *las, rhl*, and *pqs* quorum sensing systems transcription and receptor expression was highest for PA14 in NB, which had a mid-level optical density compared to PA14 in CAMHB and in DMEM FBS. The lack of increased quorum sensing for PA14 in CAMHB, which yielded the highest optical density, or in DMEM FBS, which yielded the highest virulence for select systems, suggest that increased cell density and quorum sensing are not prerequisites for increased expression of host colonization related virulence expression in *P. aeruginosa* PA14.

While increased virulence gene expression was not dependent on increased growth, our study showed that increased virulence activity correlates more closely with growth. Specifically, for T4P-mediated twitching motility, the size of *P. aeruginosa* PA14 twitching zones correlated more closely to the growth curve when the bacteria were cultured in each media. Overall, the larger twitching zones were produced when PA14 was in a broth condition as opposed to a host-mimicking condition. Serum albumin has been shown to inhibit the *P. aeruginosa* quorum sensing and to inhibit virulence factor production that would kill *S. aureus* (Smith et al., 2017). This could also explain the reduced biofilm formation of PA14 in DMEM FBS and complete absence of biofilm development of PA14 in DMEM HuS (Hammond et al., 2010). Also, twitching motility studies are traditionally performed in nutrient-rich bacterial broth conditions like the NB and CAMHB and may have contributed to the larger twitching zones. The larger twitching zones for NB and CAMHB do correlate somewhat with the biofilm development assay. While PA14 in CAMHB began biofilm development later than in DMEM 0, it increased substantially by the 8 h time point. Additionally, at 4 h, PA14 biofilm development in NB was second highest. The fact that only T4P initiation genes *FimU*, *PilW*, *PilX*, and *PilY1* were promoted, while pili elongation factor PilA and other pilin components were not significantly changed, could also contribute to the dichotomies seen in the twitching motility and biofilm formation studies (Supplementary Table 2).

Our pyoverdine virulence assessment yielded results that were most in line with our gene expression results. PA14 in DMEM HuS yielded the most pyoverdine, with PA14 in DMEM FBS also showing pyoverdine production. Previous studies have shown substantial pyoverdine production in serum-free medium under static conditions (Kang et al., 2024). We however, maintained planktonic cultures for this experiment, which is representative of antimicrobial screening conditions. From our results, serum indeed appears to promote pyoverdine production, even though iron is present. The reasons for this are currently unknown and require further study.

Overall, our study yielded compelling support for considering host-mimicking conditions to screen anti-virulence therapeutics against planktonic *P. aeruginosa*. Multiple infection-establishing virulence factors were increased under host-mimicking conditions that remained unaffected under standard AST conditions. Adequate expression of virulence targets is critical so that potentially potent and valuable anti-virulence therapeutics are not discarded in the screening stages. Knowledge of this virulence promotion, along with the dichotomy different sera produces, stands to be transformative to anti-virulence screening efforts against all MDR and XDR bacteria.

## Data Availability

The authors acknowledge that the data presented in this study must be deposited and made publicly available in an acceptable repository, prior to publication. Frontiers cannot accept a manuscript that does not adhere to our open data policies.
